# Unmasking a Rare Cause of Acute Abdomen in Adults: A Case Report of an Infected Urachal Cyst

**DOI:** 10.7759/cureus.67787

**Published:** 2024-08-26

**Authors:** Jonathan Vandeloise, Martin J Fievez, Céline Couvreur, Françoise Steenebruggen

**Affiliations:** 1 Emergency Department, Cliniques Universitaires Saint Luc, Brussels, BEL; 2 Radiology Department, Cliniques Universitaires Saint Luc, Brussels, BEL; 3 Emergency Department, Centre Hospitalier Universitaire (CHU) UCLouvain (Université Catholique de Louvain) Namur - Godinne, Godinne, BEL

**Keywords:** urachal abscess, urachus, adult urachal cyst, infected urachal cyst, acute abdomen

## Abstract

The urachus is a remnant of the fetal duct that typically obliterates and becomes a fibrous cord after birth. However, in rare cases where this process fails, urachal cysts and other abnormalities can form, often remaining undiagnosed due to their asymptomatic nature. Infection is the primary complication and can be misdiagnosed due to the cyst's obscurity and varied presentations. Delayed diagnosis can lead to severe complications such as sepsis, fistula formation, and cyst rupture, potentially causing peritonitis. This paper discusses a 48-year-old male who consulted in the emergency department with acute abdominal symptoms, was diagnosed with an infected urachal cyst via imaging and successfully treated with antibiotics and subsequent surgical excision.

## Introduction

Urachal anomalies, including urachal cysts, represent a rare group of congenital disorders resulting from incomplete obliteration of the urachus, a vestigial structure connecting the fetal bladder to the allantois [1]. While typically asymptomatic, these anomalies can present with significant clinical complications, such as infection or malignant transformation, particularly when diagnosis is delayed into adulthood. The rarity of urachal anomalies [2,3], coupled with the wide range of possible presentations [4], often presents a diagnostic challenge, particularly in the emergency department setting, where acute abdominal pain is a common complaint.

This case report details the presentation, diagnosis, and management of an adult male who presented with acute abdominal pain secondary to an infected urachal cyst. Despite the general lack of awareness and the absence of formal guidelines for the management of urachal cysts, this case emphasizes the importance of considering this diagnosis in patients presenting with unexplained abdominal pain. The report also highlights the role of imaging in diagnosing urachal anomalies and underscores the need for a multidisciplinary approach to manage these cases effectively.

Through this case, we aim to contribute to the limited body of literature on urachal cysts, providing insights into the clinical presentation, diagnostic approach, and treatment options, thereby supporting the development of a more standardized approach to managing this rare but potentially serious condition.

## Case presentation

A 48-year-old male presented to the emergency department for sharp acute abdominal pain. It was localized in the middle of the hypogastric region and was associated with the development of a small bulge. The patient did not report any discharge from the umbilicus. He reported symptoms of acute gastroenteritis three days prior, including chills, low-grade fever, muscle aches, and watery diarrhea. There were no additional symptoms, such as vomiting, chest pain, shortness of breath, hematuria, or dysuria. The patient had no previous medical history and was not taking any specific medication.

Vital signs were monitored with a body temperature of 38.3 degrees Celsius, a heart rate of 98 beats per minute, a blood pressure of 139/83 mmHg, and a respiratory rate of 14 breaths per minute, with an oxygen saturation of 98% on room air.

His skin was well-colored and warm. Cardiac and lung examinations were normal, and bowel sounds were present. Physical examination revealed a soft and non-distended abdomen, yet a tender centimetric lump was palpable on the medial line, slightly below the umbilicus, with sensitivity extending to the hypogastrium and, to a lesser extent, the left iliac region. It was not affected by Valsalva. There was no rebound or guarding.

Laboratory analysis included a urinalysis, which demonstrated slight leukocyturia and microscopic hematuria. Notable findings in the blood sample indicated hyperleukocytosis accompanied by elevated inflammatory markers, as presented in Table 1. Renal function, coagulation tests, and ion balance were found to be within the normal range.

**Table 1:** Patient’s blood biology results

Dosage	Result	Reference Range
C-reactive protein (CRP)	197.2 mg/L	< 5 mg/L
White blood cell count	17.31 x 10³/µL	4-10 x 10³/µL
Neutrophils	13.15 x 10³/µL	1.6-7 x 10³/µL
Neutrophils %	76%	40-70%

**Figure 1:**
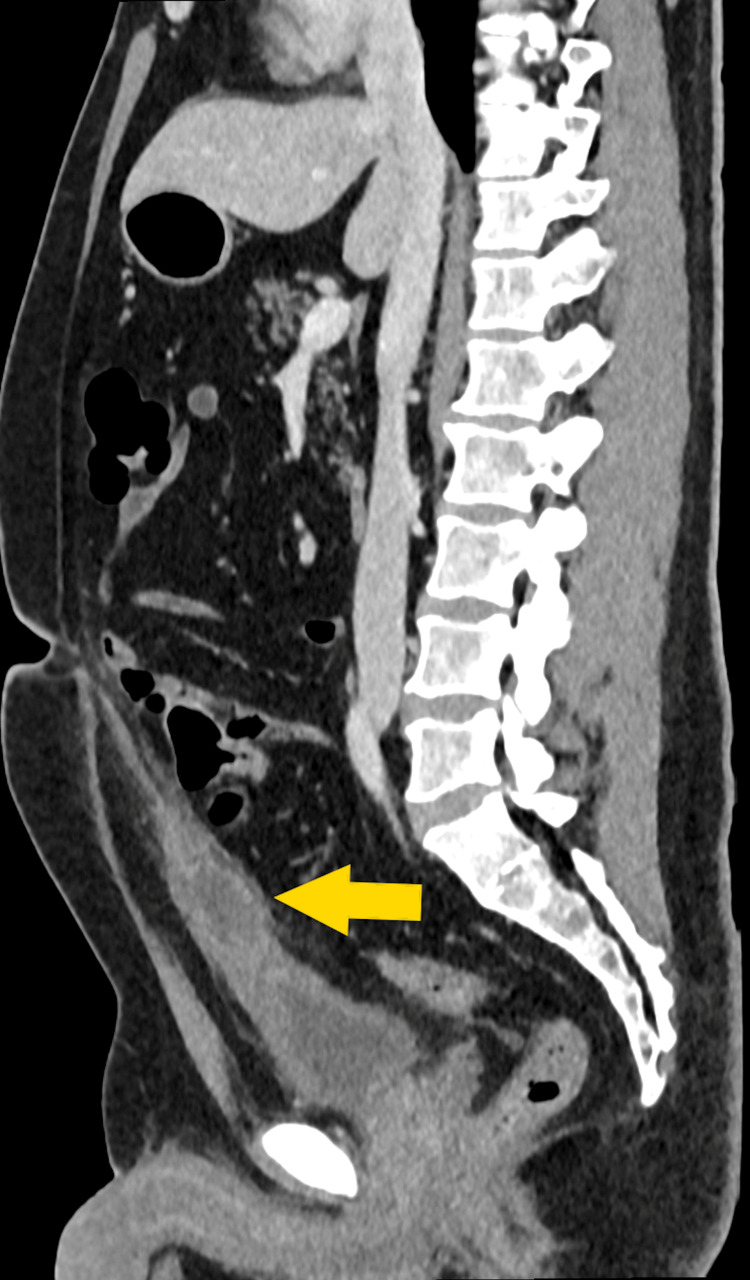
Sagittal section of the CT scan showing the urachal cyst (arrow) between the umbilicus and the bladder CT: computed tomography

A contrast-enhanced computed tomography (CT) scan of the abdomen and pelvis (midline sagittal image) was conducted (Figure 1). The scan showed thickening and increased enhancement of the bladder's wall, along with a urachal cyst featuring thickened and highly enhanced walls. Moreover, there was an infiltration of the fat surrounding the bladder and the urachal ligament, extending up to where the ligament connects to the umbilicus.

**Figure 2:**
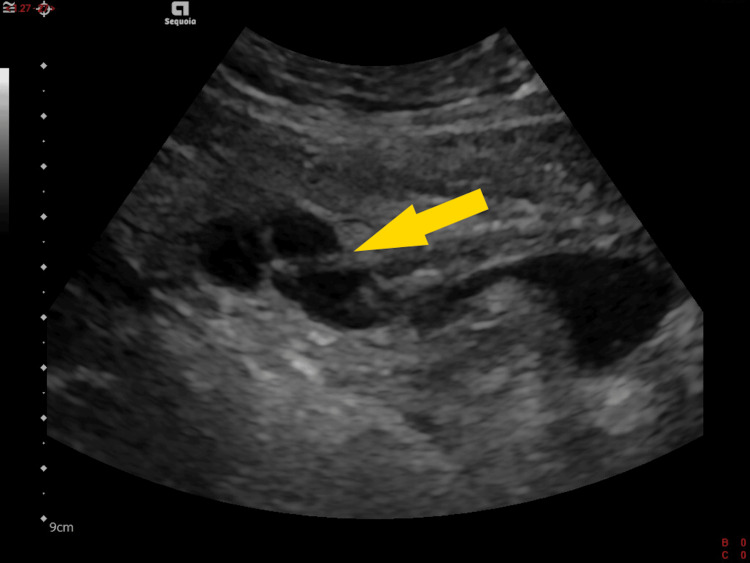
Sagittal section of US showing the urachal cyst (arrow) to be located in front of the bladder and divided into three portions as a result of the cross-sectional effect US: ultrasound

The patient was treated with oral amoxicillin-clavulanic acid 875 mg/125 mg every eight hours and discharged home. A follow-up abdominal ultrasound was performed four days later. It showed that the abscess had increased in size by 30% (Figure 2), necessitating the continuation of antibiotic therapy for a total duration of three weeks. Complete clinical and biological resolution was achieved by day 21. No other imaging tests were performed.

A cystoscopy was then performed and confirmed the absence of communication between the bladder and the cyst. Following a discussion with the patient regarding the potential risk of reinfection and carcinogenesis associated with this cyst in the future, a minimally invasive, robot-assisted excision was performed on day 71, without any further complications.

## Discussion

The urachus is a remnant of a channel between the urinary bladder and the umbilicus where urine initially drains from the fetus during the first trimester of pregnancy. The channel of the urachus usually seals off and obliterates around 12 weeks of gestation. All that is left is a small fibrous cord between the urinary bladder and umbilicus called the median umbilical ligament. A disruption of this process can result in a spectrum of rare anomalies, such as urachal cysts. They are characterized by a partial patency in the mid-duct with closure at both the umbilicus and the bladder, resulting in the formation of a non-communicating cyst [1]. The true incidence of urachal abnormalities is unknown. However, a retrospective review of a Canadian radiological database revealed that such malformations were incidentally identified in 1.03% of pediatric patients who underwent abdominal imaging studies between 2000 and 2012. Nine percent of the subjects presented with a urachal cyst [2].

While ultrasound (US) is the initial screening imaging tool in suspected cases of infection, CT and magnetic resonance imaging (MRI) are employed as definitive imaging modalities and for the evaluation of the anatomic relationship between the infected urachal remnant and adjacent structures. In addition, fluoroscopy studies can be useful. Cystoscopy is less common [5]. Some studies have indicated that urachal abnormalities may not be detected until adulthood, with a mean age of presentation of approximately 32 years [3].

The range of clinical presentation can be widely different, and patients may present with hematuria, pain, dysuria, umbilical drainage, or as an incidental finding during surgery for another disorder [4]. The cyst may become infected with gram-positive skin flora or anaerobic enteric organisms [6]. Intraperitoneal rupture of the infected cyst can result in acute peritonitis and can cause fistulization to the bowel, bladder, or skin, as well as mechanical obstruction of the gastrointestinal or genitourinary systems. Additional complications include urinary retention, hemorrhage, and an elevated incidence of adenocarcinoma (which constitutes less than one percent of all bladder cancers) [7].

In the absence of formal guidelines and in alignment with the prevailing literature, the recommended treatment for urachal abscesses in adult patients is a combination of broad-spectrum antibiotics and, if necessary, incision and drainage as initial therapy [8]. Given the potential for malignant degeneration and reinfection [9], complete excision is recommended. There are no specific guidelines for surgical management in one or two stages. Some authors have proposed a two-stage approach with antibiotics (+/- incision-drainage), followed later by surgical excision [10-12]. The two-step surgical procedure has been noted to reduce the frequency of postoperative complications [13] and was chosen for our patient.

## Conclusions

Despite the fact that infection represents the most common complication of urachal cysts, this abnormal fetal remnant remains a rare occurrence, with diagnoses often delayed until adulthood. It is therefore important to consider this when managing a patient with an acute abdomen in the emergency department. Imaging modalities, including US, CT, and MRI are of pivotal importance in the diagnosis and evaluation of urachal anomalies. The standard treatment protocol involves a combination of broad-spectrum antibiotics and surgical intervention. Complete excision is the recommended course of action to prevent recurrences and potential malignant degeneration. The case presented serves to illustrate the importance of a multidisciplinary approach to the management of urachal abnormalities. Timely recognition and appropriate intervention are key to achieving successful outcomes and patient recovery.
